# The coordination of plural logics of action and its consequences: Evidence from plural medical systems

**DOI:** 10.1371/journal.pone.0189841

**Published:** 2017-12-18

**Authors:** Jae-Mahn Shim

**Affiliations:** Department of Sociology, Korea University, Seoul, Korea; Charles Sturt University, AUSTRALIA

## Abstract

Drawing on the theory of social action in organizational and institutional sociology, this paper examines the behavioral consequences of plural logics of action. It addresses the question based on the empirical case of plural medical systems that are composed of both biomedicine and alternative medicine. Applying mixed methods of a cross-national panel data analysis and a content analysis of medical journal articles, it finds that plural systems affect health outcomes negatively when tensions between biomedicine and alternative medicine are unaddressed. In contrast, plural systems produce tangible health benefits when biomedicine and alternative medicine are coordinated through government policies or by health care organizations/professionals. This paper proposes plurality coordination as an important mechanism that modifies the behavioral consequences of plural logics. This proposition contributes to providing theoretical answers to the sociological puzzle that plural logics of action produce inconsistent behavioral consequences.

## Introduction

### Limits of the sociology of plural action logics

Logics guide behavior in several forms, such as values, habits, organizations, institutions, skills, and cultures [1–6]. Behavior is increasingly shaped by the plurality of these logics, as by multiple organizational logics [7–9], diverse institutions [10, 11], heterogeneous cultures [12–17], hybrid habits and habitus/habiti [18–20], or multiple identities [21–24]. These plural action logics are not always compatible with one another. They are often conflicting and contradictory. Thus, how social actors organize their behavior under plural logics and what behavioral consequences these logics produce are substantial sociological questions in prominent subfields [18, 25–29].

Two competing accounts exist. When plural logics are compatible with one another, more logics add up to one another and produce more resources for action, promising favorable consequences in health [22, 23], government welfare benefits [21], school lives [30], and movement mobilization [31]. When plural logics are conflicting, more logics produce strains on action, making behavior under plural logics uneasy and unsuccessful as in adolescent sexual behavior [12] and national economic policies [24, 32]. Neither account, however, explains these contradictory consequences at once.

In particular, neither of these simplistic accounts can accommodate the inconsistent consequences from plural medical systems that are composed of both biomedicine and “alternative medicine,” such as acupuncture, herbal medicine, ethnic medical traditions, and spiritual healing [33–35]. The medical literature employs a variety of terms to refer to these medical practices that are in tensions with conventional biomedicine, such as complementary and alternative medicine (CAM), complementary and integrative medicine (CIM), and traditional, complementary and alternative medicine (TCAM). Highlighted in these terms are different normative views on the relationship between conventional biomedicine and unconventional medical practices. As a way to remain neutral and thus to be ultimately able to examine the relationship in practice, this paper uses the term “alternative medicine” which may or may not be integrated into biomedicine as complementary medicine. (For the rationale to use the binary representation of biomedicine and alternative medicine, refer to the multi-dimensional tensions between biomedicine and alternative medicine elaborated right after the Introduction; for the operational conceptualization of alternative medicine to delineate the empirical data, refer to the Data sections of panel data analysis and content analysis.)

The account of additive resourcefulness in the sociological literature seems to be consistent only with the findings that plural medical systems composed of biomedicine and alternative medicine provide diverse medical resources which result in occasional treatment synergies between biomedicine and alternative medicine [36, 37] and the emotional support and empowerment of patients with alternative medical practices [19, 38, 39]. However, this additivity account does not simultaneously explain tensions between biomedicine and alternative medicine and subsequent adverse consequences, such as conflicts between treatment regimens in biomedicine and those in alternative medicine [40], confrontations between professionals of biomedicine and those of alternative medicine [41], insufficient communication between medical doctors and minority patients on alternative medicine [42], premature deaths of HIV/AIDS patients selecting alternative medicine over the biomedical drug treatment [19], and adverse interactions between medications and herbal/dietary supplements [43]. The alternative sociological account of strains on action seems to be more suitable for accommodating these findings. However, this strains account then fails to incorporate the aforementioned beneficial consequences. The critical puzzle that plural medical systems lead to inconsistent healthcare outcomes still remains to be answered.

### Limits of medical studies

Medical studies, for their part, attempt to comprehend these inconsistent outcomes with scientific medical trials [44]. Upon realizing that significant discrepancies persist even between these trials, they then use a meta-analytic approach which recalibrates the true efficacy of medical interventions by relating inconsistent outcomes to the variations in trial design, such as the characteristics of trial subjects, the medical technologies used, and the outcome measures [45, 46]. While resolving some of the inconsistent outcomes, the meta-analytic approach is still perplexed by the remaining inconsistencies between trials of an identical design. These remaining inconsistencies seem to be related to the geographical location of trials [47] or the ways in which alternative medicine interventions are arranged with the surrounding biomedical interventions [48].

However, medical trials and meta-analyses rarely investigate how users and practitioners behave–and are indeed conditioned to behave–at the intersection of the two different logics of medical practices: biomedicine and alternative medicine. The wisdom that the treatment effect of biomedical interventions is significantly modified by indigenous medical cultures [14, 49] has not spawned the notion that the effect of alternative medicine can be conditioned by biomedical treatment environments. This paper fills in this deficiency and further develops this notion by embedding it to the sociology of organizations and institutions that goes beyond the two preceding simple accounts of plural action logics.

### Re-engaging the sociology of plural action logics

The sociological literature shows that even conflicting plural logics are organized to co-exist with one another. First, they are organized through the individual wisdom and skill of actors, as in skillful workmen with a toolkit of diverse cultural models during settled lives [13], artful individuals and organizations managing institutional conflicts in work–family relations [50] and democracy–bureaucracy relations [10], innovative government actors conjuring novel institutions by patching institutions of diverse national origins [51], creative LGBT movement organizers anchoring unrelated and divisive movement motives in the most common social movement ideas [7], life scientists constructing a hybrid exchange logic between the conflicting logics of science and commerce by redefining the meaning and practices of patenting [11], museum professionals progressively upholding the conflicting goals of conservation and exhibition [8], and lay people aptly appropriating foreign medical elements into the indigenous medical system [52].

Second, supra-individual forces guide conflicting logics to be organized and co-practiced, such as the historical-structural conditions surrounding individuals with unwieldy tools during unsettled periods [13]. The medical habitus of South Africans who co-practice the incongruent elements of biomedicine and traditional African medicine is guided structurally by the forces of late global capitalism [19]. In addition, the hybrid medical habitus of U.S. immigrants, who often rely on ethnic/folk medicine along with biomedicine, is directed by collective forces, such as ethnicity and social ties [18].

While informative, these studies still fall short of a theory of the behavioral consequences of plural logics. They do not examine the consequences of plural logics and, instead, only demonstrate the organizability of conflicting logics. It remains unexamined whether plural logics produce any difference in behavioral outcomes when they are organized compared to when they are not. For example, it is unclear how the hybrid logic of exchange developed by skilled scientists [11] changes their practice of science, compared to when there is no such hybrid logic. Likewise, it is unexamined what kinds of differences the “thin” [15] and “limited” [16] anchoring of divergent movement ideas in the most general causes (e.g. community-building and equality) brings to the LGBT movement [7], compared to when there is no such anchoring. Art museums’ and museum professionals’ performance during the brief co-existence of the conflicting goals of conservation and exhibition is not examined against their performance under the single goal of exhibition [8].

Meanwhile, some of these studies provide intriguingly contradictory observations on the consequences of organized plural logics. For example, the plural health behavior of HIV/AIDS patients in Africa produces prolonged lives in some countries [38, 53] and premature deaths in others [19], all of which are situated in the same structural condition (late global capitalism). While the medical habitus utilizing both biomedicine and alternative medicine commonly produces psychological relief and empowerment [19, 39], one case leads to premature deaths [19] while the other shows no clear impact on lives [39]. However, these substantial contrasts, such as the winning-vs- losing hybrid habitus, the winning-vs-losing organization of plural logics, and the quick/easy–vs–slow/difficult organization of logics, have not been theorized within a single conceptual frame yet.

A closer examination even reveals that conflicting logics are in fact organized only among some actors and not others. For example, the appropriation of the meaning of patenting is observed unevenly among life scientists. Thus, the subsequent organization of conflicting logics of science and commerce is found to be relatively easier among “senior” scientists than “juniors” [11]; the organization is easier for scientists who often interact with other professionals in law and administration than those who do not [11]. Different logics of medical practices, such as biomedicine and traditional African medicine, are organized with the help of medical care professionals only in some countries [38, 53] and not in others [19]. The organizability and the locus of organizing agency during “settled” periods differ from those during “unsettled” periods [13]. Museum professionals’ progressive upholding of the conflicting goals of conservation and exhibition seems to be tenable when there is sufficient budgetary support, whereas this is easily threatened by budgetary constraints [8].

Thus, this paper purports to illuminate that some conflicting plural logics are coordinated and produce favorable consequences while others are hardly coordinated and result in undesired consequences. The paper argues that conflicting plural logics produce favorable consequences when they are coordinated within supportive social contexts. Otherwise, it is difficult for individuals to organize conflicting logics and utilize them to their benefits. The paper bases this argument on the varying and even contradictory health outcomes under the plural medical systems of biomedicine and alternative medicine. A cross-national panel data analysis tests two hypotheses about the varying health outcomes of plural medical systems. Then, a comparative analysis of the U.S. and Japan demonstrates how results from the cross-national panel analysis are triangulated by medical treatment episodes in medical journals.

### Plural medical systems and varying health consequences

Medical practices are found to be pluralist for economic, cultural, and medical reasons in the developed as well as developing world [33, 54, 55]. A majority of countries have official policies on popular alternative medicine practices, such as Ayurveda, Chinese medicine, homeopathy, osteopathy, chiropractic, bone-setting, herbal medicine, and spiritual/religious therapies [33]. About half of these countries have government financial provision for alternative medicine. Alternative medicine is utilized widely to treat various medical conditions, such as infectious diseases (e.g. HIV/AIDS) and chronic health problems (e.g. cancers, cardiovascular conditions, and musculoskeletal problems). Therefore, its impact can be consequential for national health outcomes across the world in a positive or negative way.

The additivity account of plural logics posits that plural logics linearly add up to the resourcefulness of strategies for action. Thus, a plural medical system provides more resources to manage health and promises better health outcomes than a system of biomedicine alone. This resourceful additivity account finds any adverse health outcomes to be unexpected and attributable only to the invalidity of alternative medicine for health management [44]. On the other hand, the strains account of plural logics suggests that a plural medical system is laden with conflicts between biomedicine and alternative medicine. Adverse health outcomes from the conflict–loaded plural medical system are inevitable. These adverse outcomes are not prevented by simply improving the safety and efficacy of alternative medicine. On the contrary, any desired positive health outcomes are viewed as unexpected consequences. Challenging is not only the conceptual bifurcation of these two accounts but also the discrepancy between each account and empirical evidence. To address these deficiencies, this paper formulates an alternative account in which tensions between biomedicine and alternative medicine are not overlooked nor deemed insurmountable.

Plural medical systems are indeed laden with tensions between biomedicine and alternative medicine and, for this reason, produce difficulties for medical practices among lay users and professional practitioners. First, these tensions are medical-theoretical. In terms of etiology, alternative medicine views a human being in its totality within a wide and remote ecology, and attributes ill health to the disequilibrium of this total ecological system vis-à-vis biomedicine’s emphasis on proximate causative agents and their pathogenic evolution within the boundaries of a body [56, 57]. From a social scientific approach that views medicine as a social systemic process of allocating accountability for individual and collective failures/successes, alternative medicine features a holistic and spiritual allocation of accountability, whereas biomedicine relatively centers on a partial and physical allocation [58]. Reflecting this view, the system of MeSH terms underlying the indexing in the PubMed database specifies a subset of MeSH terms that group together a variety of medical practices as complementary and alternative medicine (for detailed strategies, refer to http://www.nlm.nih.gov/bsd/pubmed_subsets/comp_med_strategy.html) [59].

Second, the tensions are political and cultural as well. In the social constructionist view of medicine and medicine-as-profession [60, 61], any brand of medicine employs the process of political and cultural negotiation. Biomedicine is historically negotiated as science and progressive modernity, whereas alternative medicine is portrayed as non-science, magic, and a backward tradition [62–64]. In colonial and postcolonial contexts, biomedicine is aligned with global domination projects, whereas alternative medicine is often identified with local autonomy [19, 65].

These medical-theoretical and political-cultural tensions are evident in the behavior of medical service users and professional practitioners. Cancer patients who use both biomedicine and alternative medicine are often caught up between two conflicting theories, or biomedicine’s depersonalized approach and alternative medicine’s individualized approach [66]. When patients reclaim their control over medical decision-making through the use of alternative medicine, they are not always confident about their knowledge base and are often dependent on the advice of biomedical professionals [67]. Patients’ orientation toward spiritual healing comes into conflict with physicians’ treatment regimens [68, 69]. Users of alternative medicine do not readily discuss their use of alternative medicine with their physicians, since physicians are often found to ignore alternative medicine [70, 71].

These tensions between biomedicine and alternative medicine, however, do not create the same difficulties in different plural medical systems. Health behaviors and outcomes vary widely, depending on how these tensions are articulated in the system. For instance, national political contexts heighten or mitigate these tensions, producing different consequences. Post-Apartheid nationalist politics in South Africa, that portray alternative medicine as an indigenous African tradition and biomedicine as “Western White” science, is accused of strengthening inter-professional barriers in HIV treatment between medical doctors who provide drug therapy and traditional healers who practice herbal and spiritual healing [19]. It is also blamed for creating unnecessary social criticism on HIV patients that prevents them from taking an effective biomedical drug therapy (ART). In other African countries (e.g. Uganda, Kenya, and Tanzania) whose political environments promote alternative medicine education for biomedical practitioners and the cross-referral of patients between biomedicine and alternative medicine practitioners, HIV patients take the drug therapy for controlling the viral loads and, simultaneously, use herbs and spiritual healing for their immune systems and emotional well-being [38, 53].

Micro-level therapeutic environments matter, as well. For instance, Steve Jobs spent the first nine months of his diagnosis with pancreatic cancer trying alternative medicine, such as diets, fruit juices, acupuncture, herbal remedies, and spiritual practices [72]. Disagreement over his behavior aside, his personal account exemplifies that alternative medicine users outside institutionalized settings often go through difficult responses from families, friends, and even doctors who are “infuriated” and “distressed” by their reliance on alternative medicine. Thus, patients often have to “search the Internet” to get information about alternative medicine. The use of alternative medicine becomes a lonely and secretive process. In another context as in integrative cancer care centers, however, plural medical practices are open and communicative between patients, families, and practitioners [73, 74].

### Hypotheses and two complementary analyses

Therefore, this paper hypothesizes that tensions between biomedicine and alternative medicine and their management in institutional settings are consequential for health behavior [75, 76] and outcomes [59]. It conducts two complementary analyses: one at the macro level with cross-national panel data, testing two hypotheses on the changing relationships between plural medical systems and national health outcomes; the other at the micro level with data extracted from medical journal articles in the U.S. and Japan, elaborating how macro-level patterns are related to the treatment-level evidence.

The macro-level analysis takes two steps. First, it examines how the plurality of a national medical system affects health outcomes. It is hypothesized that plurality will affect health outcomes negatively as tensions between biomedicine and alternative medicine produce strains on the behaviors of users and practitioners (*Hypothesis 1*). Strains on health behaviors are identifiable in the delayed utilization of relevant medical resources, whether they are biomedicine or alternative medicine [77, 78], the non-utilization of these relevant resources [58, 79], and the ill-informed/ill-sequenced combinations of biomedicine and alternative medicine that lead to adverse interactions between the interventions [80]. Delayed utilization, non-utilization, and ill-informed utilization of medical resources, in turn, affect health care outcomes adversely.

Second, this paper examines how measures that ameliorate tensions between biomedicine and alternative medicine change the relationship between plurality and health outcomes. It hypothesizes that measures of tension reduction will weaken the negative effect of plurality and convert it into a positive one, since reduced tensions help resolve the strains of plural logics and generate their resourcefulness through treatment synergies between biomedicine and alternative medicine (*Hypothesis 2*).

In particular, the paper examines government funding for alternative medicine and medical knowledge production as two promising measures of coordination mechanism that reduces the tensions. Government funding for alternative medicine refers to the existence of public financial support for alternative medicine through government insurance coverage or free provisions of alternative medicine in public health facilities. Either through direct service provisions or rules for insurance reimbursement, governments provide specific ways to practice alternative medicine along with biomedicine, thus reducing biomedicine–alternative medicine tensions and adverse outcomes. Medical knowledge production refers to the annual number of medical journal articles produced by each country. It measures the extent to which information on medical interventions is circulated within the medical community. In countries producing more papers, the communication between biomedicine and alternative medicine is greater and helps inform medical practitioners on both sides, thus lowering professional barriers between the two sides.

The second-step micro-level analysis conducts a content analysis to elaborate different ways to coordinate plural medical systems and the extent to which plurality coordination moderates the effects of plural medical systems [81] in the U.S. and Japan. This analysis reveals the medical treatment-level evidence on biomedicine–alternative medicine tensions, their institutional environments, and treatment outcomes. Subsequent multivariate regression models of the results of this content analysis ultimately demonstrate that cross-national differences in the treatment outcomes are explained by cross-national differences in biomedicine–alternative medicine tensions and their coordination.

This analysis examines the U.S. and Japan as a comparative pair, since they feature relatively pluralist medical systems [33, 56] and provide a clear contrast in the institutionalization of the plural systems [59]. The U.S. features a market-based decentralized and informal coordination of biomedicine and alternative medicine [82, 83], whereas Japan represents a government-driven centralized formal coordination [84, 85]. This contrast makes it promising to examine how different institutional settings are aligned with treatment outcomes.

## Analysis 1: cross-national panel data

### Data and measures

This paper uses an unbalanced panel dataset of 246 observations for 97 countries for three time points (S1 File). The dependent variable, national health outcomes, is measured by *life expectancy at birth* in 1995, 2000, and 2005 [86]. Independent variables, including the key variable of medical plurality, are measured five years earlier for these years, in order to allow the delay in time when changes in independent variables are reflected in the dependent variable.

*Medical plurality* is defined as the extent to which a national medical system is composed of two different elements: biomedicine and alternative medicine. To quantify this quality, this paper adopts the idea commonly applied to measuring racial/ethnic diversity [24, 87]. The literature quantifies societal diversity with the following formula:

$$\begin{array}{c} \frac{1-\frac{\sum n_{i}(n_{i}-1)}{N(N-1)}}{1-\frac{1}{K}}\\ \left(where\;N:\;total\;population,\;K:\;the\;number\;of\;subgroups,n_{i}:\;population\;in\;subgroup\;i\right) \end{array}$$

It refers to the probability that two individuals chosen randomly and independently from the total population do not belong to the same racial/ethnic subgroup. The minimum value 0 indicates that no pair of individuals belongs to different subgroups; the maximum value 1 indicates that every random pair is composed of individuals from two different subgroups.

This paper defines a *medical plurality index* with the following formula. Since the number of subgroups (K) is 2, the preceding formula becomes simplified and refers to the probability that a randomly chosen pair of medical resources belong to two different subgroups: biomedicine (n_*1*_) and alternative medicine (n_*2*_).

$$\begin{array}{c} \left[1-\frac{\sum n_{i}(n_{i}-1)}{N(N-1)}\right]\times 2\\ \left(where\;i=1,2\right) \end{array}$$

The amounts of biomedical resources (n_*1*_), alternative medicine resources (n_*2*_), and total medical resources (N = n_*1*_ + n_*2*_) are approximated from the medicine section of the Yearbook of International Organizations [88]. This yearbook lists international professional medical organizations with their member states and organizational aims/activities. Thus, it is possible to compute for each country the number of memberships in biomedicine organizations (n_*1*_), the number of memberships in alternative medicine organizations (n_*2*_), and their sum (N) (for further detail, see Text 1 in S2 File and Table A in S2 File).

The mechanism of plurality coordination that reduces tensions between biomedicine and alternative medicine is first measured by *government funding for alternative medicine* [33, 89]. It is an indicator variable coded 1 if there is a government financial provision in any of the following three manners: (1) government insurance coverage of alternative medicine therapies; (2) free-of-charge provisions of the therapies in public health facilities; (3) other public financial support.

The second measure of plurality coordination is examined in terms of *medical knowledge production*. It measures the total number of medical journal articles that each country produces per year. To get this measure, this paper links medical journal articles indexed in the U.S. National Library of Medicine’s MEDLINE database to authors’ institutional affiliation data in Thomson Reuters’ Web of Science [90]. By tagging the geographical information of author institutions to papers, it assigns each of these papers to one or more countries.

In order to specify the effect of the medical plurality index, net of the impacts of the amounts of different medical organizational memberships, this paper controls for *alternative medicine organizational membership rate*, *biomedicine organizational membership rate*, and *the total organizational membership rate*. These are computed by the counts of organizational memberships divided by national population (per million people). With these control variables, this paper distinguishes the effect of medical plurality from that of the amount of medical resources approximated in the numbers of organizational memberships in biomedicine, alternative medicine, and both.

This paper controls for additional variables, such as economic development [91], education [92], and income inequality [93]. Economic development is measured by *GDP per capita*, education by *the average total years of schooling* for people aged 25 or more [94], and income inequality by *the standardized Gini coefficients of income* [95].

### Models

Under the panel data structure, the random effects model (REM) and the fixed effects model (FEM) are used [96]. In order to adjust for the unmeasured temporal changes, the models incorporate two dummy variables for 1995 and 2000 against 1990.

*Hypothesis 1* is tested by:

$$\begin{array}{l} {Life\;Expectancy}_{i(t+5)}\\ \;=Year\;Dummies+{Medical\;Plurality\;Index}_{it}\\ \;+{Amounts\;of\;Medical\;Resources}_{it}+{Socioeconomic\;Conditions}_{it}\\ \;+a_{i}+\varepsilon_{i(t+5)} \end{array}$$ (Eq 1)

where *i* indicates each country; *t* indicates year; *a*_*i*_ indicates a time-constant unit-specific effect for country *i*; *ɛ*_*i(t+5)*_ is a random error term for country *i* in year *t+5*. This equation aims to specify regression coefficients for *Medical Plurality Index*_*it*_.

To test *Hypothesis 2*, this paper adds interaction terms between the medical plurality index and the measures of plurality coordination whose regression coefficients are the analytical focus:

$$\begin{array}{l} Life\;Expectancy_{i\left(t+5\right)}\\ \;=Year\;Dummies+Medical\;Plurality\;Index_{it}\\ \;+Amounts\;of\;Medical\;Resources_{it}+Plurality\;Coordination_{it}\\ \;+Medical\;Plurality\;Index_{it}\times Plurality\;Coordination_{it}\\ \;+Socioeconomic\;Conditions_{it}+a_{i}+\varepsilon_{i\left(t+5\right)} \end{array}$$ (Eq 2)

FEM estimates are reported (for REM estimates, see Table B in S2 File and Table C in S2 File). Statistically, the Hausman test reports significant differences between FEM estimates and REM estimates. This is reasonable because estimates in FEM and REM tend to be different in a dataset with a few time points like the dataset of this paper, whereas they tend to become similar with more time points. FEM estimates are preferred for several reasons. Since my units of analysis–i.e. countries–are hardly a random sample drawn from a larger population, it makes sense theoretically to interpret “each as a separate intercept” for each country, rather than a random variable [96]. In addition, FEM estimates do not rely on REM’s stricter assumption of the independence between independent variables and unobservable country-specific effects.

### Results

**Fig 1:**
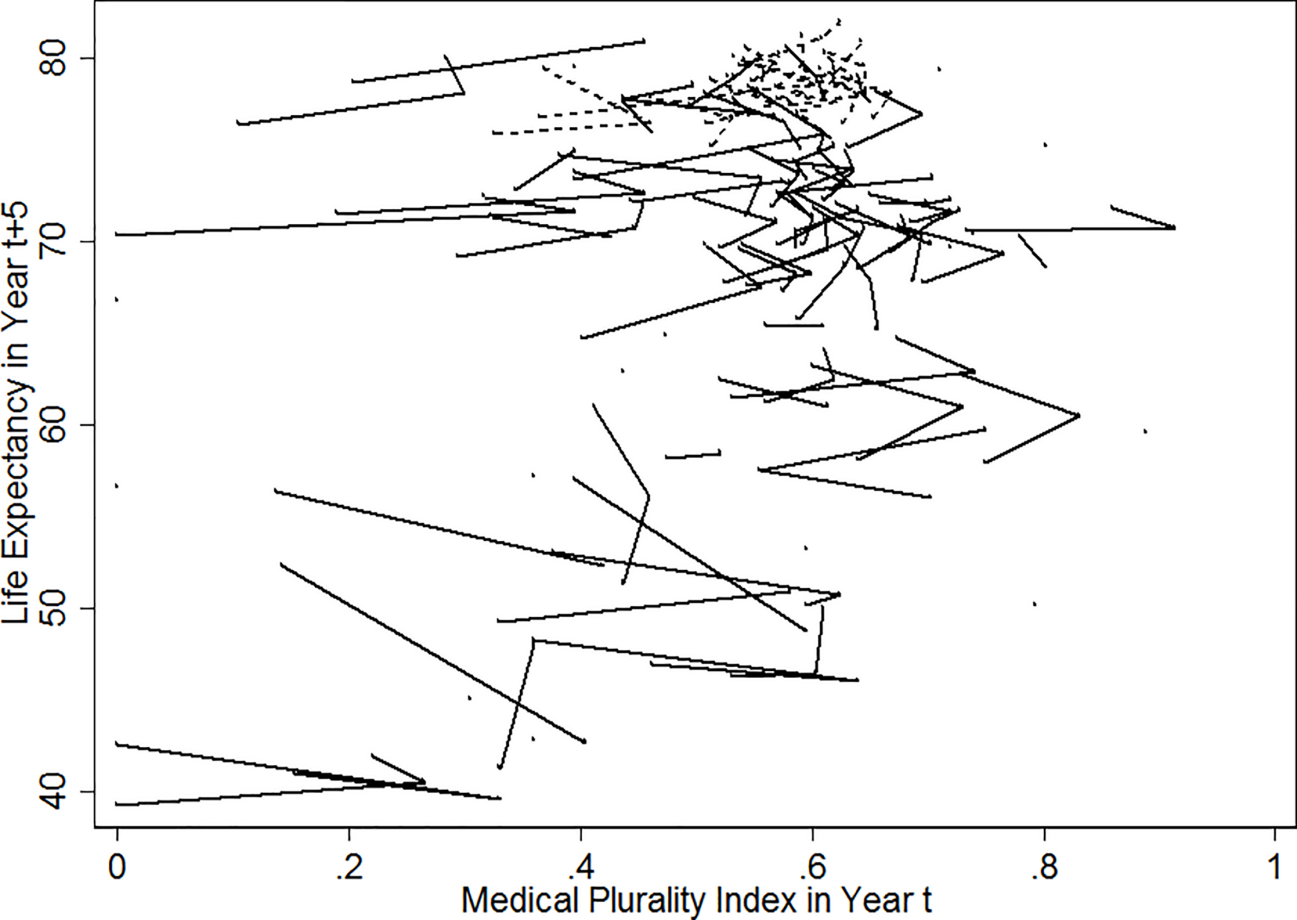
Historical scatterplot of life expectancy at birth over medical plurality index across years with lines connecting observations of a country: 246 observations for 97 countries across 1990, 1995, and 2000 (dotted lines for 18 OECD countries; solid lines for the others).

Fig 1 graphically represents how life expectancy at year *t+5* changes as the medical plurality index at year *t* (= 1990, 1995, and 2000) changes. Moving from bottom to top, each line refers to a country’s trajectory in the past five years. Positive slopes mean that life expectancy increases as medical plurality index increases. Negative slopes refer to the opposite development. It is certain in this historical representation that the relationships between medical plurality and life expectancy are not unidirectional. The first model specification (*Eq 1*) aims to find a dominant relationship, positive or negative, whereas the second (*Eq 2*) is geared to identify conditions in which the dominant relationship changes into the other direction.

The FEM estimates for *Eq 1* (Table 1) show that medical plurality has a negative effect on life expectancy (Model 1). Against the suspicion that this negative effect is simply confounded by the negative effect of the allegedly invalid alternative medicine and the positive effect of the allegedly valid biomedicine, the independent effects of biomedicine and alternative medicine are further controlled in Models 2 to 4. The negative effect of medical plurality still remains significant in these models. It remains so with even more controls in Models 5 to 8.

**Table 1:** Unstandardized coefficients from the fixed effects models of life expectancy regressed on medical plurality index and control variables.

	Model 1	Model 2	Model 3	Model 4	Model 5	Model 6	Model 7	Model 8
Year Dummies (Reference = 1990)								
1995	1.29**	1.23**	1.19**	1.22**	1.35**	1.37**	1.32**	1.36**
	(0.18)	(0.19)	(0.20)	(0.19)	(0.27)	(0.28)	(0.28)	(0.28)
2000	2.38**	2.24**	2.18**	2.22**	2.39**	2.43**	2.34**	2.42**
	(0.21)	(0.29)	(0.30)	(0.29)	(0.45)	(0.49)	(0.50)	(0.49)
Medical Plurality Index (MPI)	-5.75*	-5.58*	-6.31**	-5.65*	-5.19+	-5.25+	-5.41+	-5.20+
	(2.35)	(2.35)	(2.40)	(2.33)	(2.71)	(2.71)	(2.90)	(2.71)
Control Variables: Level of Organizational Memberships								
Biomedicine Organizational Memberships		0.05				-0.02		
(per million people)		(0.04)				(0.04)		
A.M. Organizational Memberships			0.45				0.15	
(per million people)			(0.27)				(0.28)	
Total Organizational Memberships				0.05				-0.01
(per million people)				(0.03)				(0.04)
Control Variables: Socio-economic Conditions								
GDP per capita (in hundreds)					0.01*	0.01+	0.01+	0.01+
					(0.01)	(0.01)	(0.01)	(0.01)
Standardized Gini Coefficient of Income					0.004	0.004	0.003	0.004
					(0.06)	(0.06)	(0.06)	(0.06)
Years of Education					-0.46	-0.48	-0.44	-0.47
					(0.82)	(0.83)	(0.83)	(0.83)
Constant	71.3**	70.8**	71.0**	70.8**	72.4**	72.5**	72.4**	72.5**
	(1.29)	(1.30)	(1.22)	(1.28)	(5.17)	(5.28)	(5.20)	(5.26)
Observations	246	246	246	246	246	246	246	246
Number of Countries	97	97	97	97	97	97	97	97
R-squared	0.40	0.40	0.41	0.40	0.42	0.42	0.42	0.42

*Note*: Robust standard errors in parentheses

+ significant at 10%

* significant at 5%

** significant at 1% (two-tailed tests).

This result supports the notion in *Hypothesis 1* that actors are not presumably skilled to handle conflicting logics, such that plural logics produce strains on health behaviors and result in the ill-informed or limited utilization of available plural medical resources. Through users’ ill-informed utilization or non-utilization of available plural resources, plurality affects health outcomes negatively. In Model 8, one standard deviation increase in medical plurality index (0.15) leads to a decrease of 0.75 years in life expectancy.

This identification of the dominant pattern needs to be complemented with an additional account for positive lines in Fig 1. This is achieved by *Eq 2* (Table 2) that specifies conditions in which the dominant negative effect of plurality changes into a positive one.

**Table 2:** Unstandardized coefficients from the fixed effects models of life expectancy regressed on interaction variables with medical plurality index and control variables.

	Model 1	Model 2	Model 3	Model 4	Model 5^b^^)^	Model 6^b^^)^
Year Dummies (Reference = 1990)						
1995	1.203**	1.323**	1.236**	1.412**	1.254**	1.427**
	(0.201)	(0.294)	(0.203)	(0.294)	(0.213)	(0.352)
2000	2.223**	2.383**	2.233**	2.495**	2.375**	2.594**
	(0.298)	(0.506)	(0.312)	(0.513)	(0.309)	(0.595)
Medical Plurality Index (MPI)	-6.045*	-5.647+	-5.716*	-5.323+	-9.903**	-8.862+
	(2.470)	(2.899)	(2.387)	(2.761)	(3.702)	(4.664)
Control Variables: Level of Organizational Memberships						
Total Organizational Memberships	0.054	-0.017	0.055	-0.034	0.040	-0.016
(per million people)	(0.035)	(0.038)	(0.037)	(0.046)	(0.033)	(0.034)
OECD18 Dummy^a^^)^						
(1 for OECD18 countries; 0 for else)						
MPI × OECD18 Dummy	6.236*	6.253+				
	(3.045)	(3.489)				
Number of Medical Journal Papers (in thousands)			-0.050	-0.095		
			(0.059)	(0.078)		
MPI × Number of Medical Papers			0.079	0.117		
			(0.092)	(0.120)		
Government Funding for A.M.^a^^)^						
(1 for funding; 0 for else)						
MPI × Funding for A.M.					10.174*	8.895+
					(4.243)	(5.033)
Control Variables: Socio-economic Conditions						
GDP per capita (in hundreds)		0.019*		0.023*		0.014
		(0.010)		(0.011)		(0.010)
Standardized Gini Coefficient of Income		0.004		0.005		-0.018
		(0.065)		(0.066)		(0.082)
Years of Education		-0.436		-0.529		-0.424
		(0.850)		(0.850)		(0.973)
Constant	70.290**	71.699**	70.885**	72.753**	71.374**	73.902**
	(1.081)	(5.301)	(1.313)	(5.312)	(1.033)	(6.764)
Observations	246	246	246	246	203	203
Number of Countries	97	97	97	97	76	76
R-squared	0.41	0.43	0.40	0.42	0.52	0.54

*Note*: Robust standard errors in parentheses

+ significant at 10%

* significant at 5%

** significant at 1% (two-tailed tests)

^a)^ Time-invariant indicator variables for countries are automatically dropped out of the FEM models

^b)^ It is based on a subset of 203 observations with data on government funding for alternative medicine (A.M.).

When I specify all models in Tables 1 and 2 among this subset of 203 observations, the findings agree with those reported here. Results for the subset are reported in Table D in S2 File and Table E in S2 File).

In Fig 1, positive slopes exist more among countries with high life expectancies than those with low life expectancies. Most cases with high life expectancies are affluent countries. Indeed, the estimate of the interaction term between medical plurality and OECD 18 dummy in Model 1 indicates that the effect of medical plurality on life expectancy is positive among the 18 OECD countries and negative among the non-OECD countries. Interestingly, this positive OECD effect remains the same even when socio-economic conditions are controlled (Model 2). As hypothesized, factors other than these socio-economic control variables explain the difference in the remaining four models in Table 2.

Models 3 and 4 reveal a positive interaction effect between medical knowledge production and medical plurality. As countries produce more medical research papers, the negative effect of medical plurality on life expectancy decreases and, finally, converts into a positive one. It seems that medical knowledge production plays the role of plurality coordination. Since the interaction effect is not statistically significant, however, *Hypothesis 2* cannot be univocally supported based solely on this measure.

Models 5 and 6, on the other hand, support the coordination hypothesis at a statistically significant level. The models report a significant positive interaction effect between government funding for alternative medicine and medical plurality. Government funding for alternative medicine is an important coordination mechanism through which the negative effect of medical plurality on life expectancy turns positive. In Model 6, one standard deviation increase in medical plurality index (0.15) leads to a 0.005 year gain in life expectancy among countries with government funding. On the contrary, the same increase in medical plurality leads to a 1.32 year loss in life expectancy among countries without such funding. The policy contexts in which medical plurality is practiced make such a striking difference.

## Analysis 2: content analysis comparing the U.S. and Japan

### Data and measures

Content analysis purports to elaborate tensions between biomedicine and alternative medicine, plurality coordination, and their impacts on health care outcomes. It uses a prominent medical journal database MEDLINE [97] to collect treatment-level evidence of alternative medicine in different institutional settings in the U.S. and Japan.

**Fig 2:**
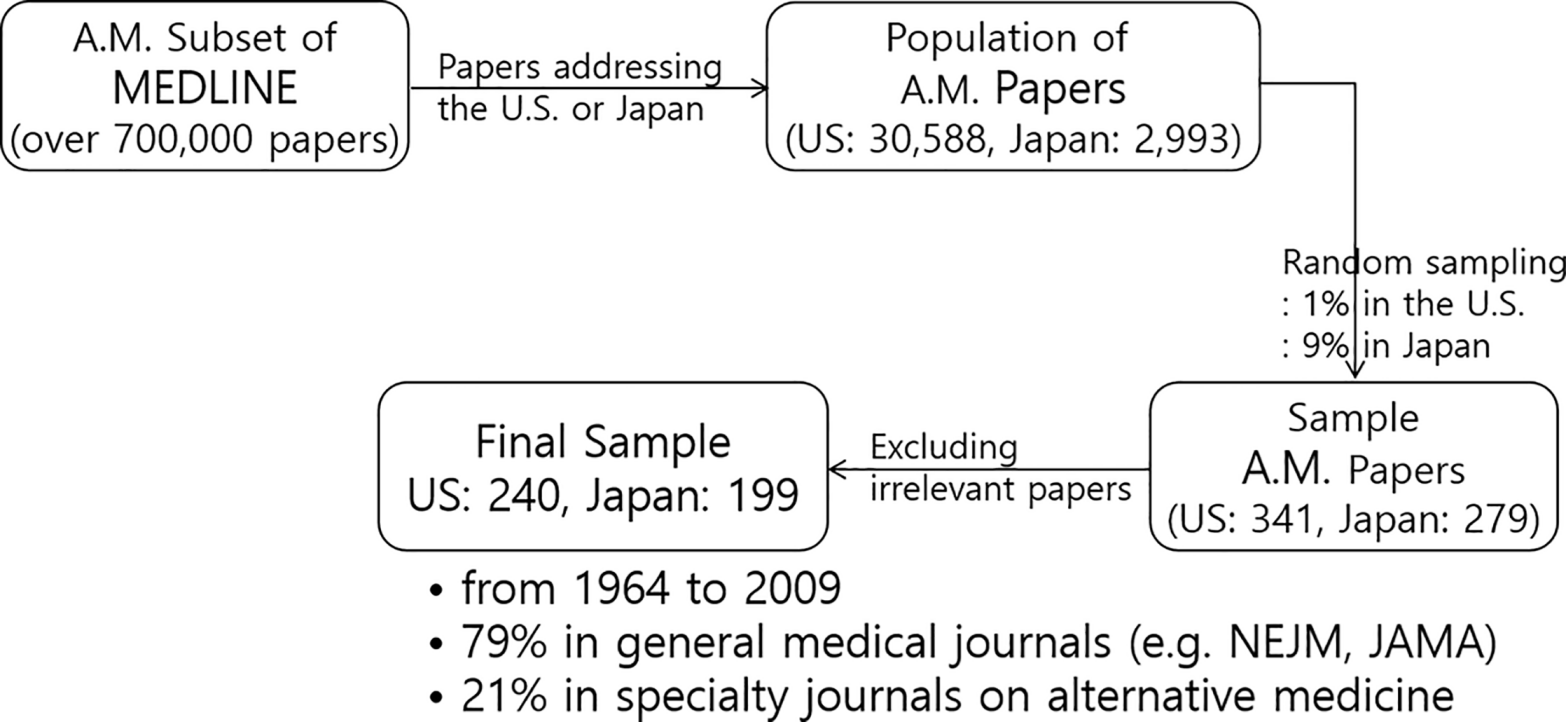
Sample selection process for the content analysis of medical journal papers.

Using search terms in the Medical Subject Headings (MeSH), such as “the United States” and “Japan,” this paper retrieves 30,588 papers based on trials in the U.S. and 2,993 papers based in Japan from MEDLINE’s subset of alternative medicine (http://www.nlm.nih.gov/bsd/pubmed_subsets/comp_med_strategy.html). Then, it randomly samples 341 papers in the U.S. (1.1% of the total) and 279 papers in Japan (9.3% of the total) for content analysis. 29.6% of the sample in the U.S. (101 papers) and 28.7% of the sample in Japan (80 papers) are subsequently excluded because they are actually unrelated to alternative medicine. 56 more papers in the U.S. and 22 more papers in Japan are excluded, because they employ multiple outcome measures and report inconsistent results. This has led to a final sample of 184 papers from the U.S. and 177 from Japan (Fig 2).

Using a paper as the unit of analysis, each paper is coded regarding what kind of alternative medicine intervention is used, for what medical conditions, and whether the intervention is found effective in treating the medical conditions. It is also coded whether there are biomedicine–alternative medicine tensions in the reported behaviors of users or practitioners, coordination efforts to reduce these tensions, and adverse-vs-synergic treatment interactions between biomedicine and alternative medicine interventions (S3 File).

### Results

Compared to Japan, alternative medicine in the U.S. is found to be less coordinated with biomedicine. Correspondingly, alternative medicine in the U.S. is found less effective, more in tension with biomedicine, and less synergic with biomedicine. In detail, the percentage of papers identifying a deficiency of plurality coordination is much higher in the U.S. (17.9% vs. 2.8%; p < 0.01). On the contrary, the percentage of papers that verify the treatment effectiveness of alternative medicine is lower in the U.S. than Japan (42.4% vs. 60.5%; p < 0.01). Tensions between biomedicine and alternative medicine are reported more often in the U.S. (6.5% vs. 0.6%; p < 0.01). So are adverse interactions between biomedicine and alternative medicine (12.0% vs. 0.6%; p < 0.01). Synergic interactions are found less often in the U.S. (3.3% vs. 5.7%; p = 0.27). Across the U.S. and Japan, papers reporting that alternative medicine is not coordinated with biomedicine are less likely to report effective treatment outcomes from alternative medicine.

The multivariate analysis of these results (Table 3) also verifies this relationship between the deficiency of plurality coordination and the treatment outcomes of alternative medicine. In particular, models in Table 3 specify 1) the U.S.–Japan difference in the effectiveness of alternative medicine treatments and 2) the extent to which this difference is explained by the difference in plurality coordination.

**Table 3:** Log odds ratios from the Logit models of the effectiveness of alternative medicine^a^^)^ regressed on the deficiency of plurality coordination and control variables.

	Model 1	Model 2	Model 3
Location of Study^b^^)^	0.731**	0.567*	0.544+
(1 = Japan, 0 = US)	(0.214)	(0.221)	(0.293)
Plurality Coordination Deficiency^c^^)^		-1.301**	-2.117**
(1 = Reported, 0 = Not reported)		(0.422)	(0.511)
Control Variables			
Journal of Publication^d^^)^			0.914*
(1 = Alternative medicine journal, 0 = General medical journal)			(0.368)
Study Design^e^^)^			2.490**
(1 = RCT, 0 = Otherwise)			(0.792)
Modality^f^^)^ (Reference = Mind-Body Therapies)			
Alternative Whole Medical Systems			1.574**
			(0.525)
Biologically Based Modalities			2.636**
			(0.397)
Manipulation-based Modalities			3.649**
			(0.741)
Other			3.074**
			(0.410)
Constant	-0.307*	-0.106	-2.568**
	(0.149)	(0.161)	(0.368)
Observations (N of Papers)	361	361	361

*Note*: Standard errors in parentheses.

+ significant at 10%

* significant at 5%

** significant at 1%.

^a)^ A binary variable coded 1 if alternative medical treatment is found effective in the paper and 0 otherwise.

^b)^ And indicator coded 1 for the paper based on trials in Japan and 0 for the U.S.

^c)^ An indicator coded 1 if an occasion of coordination deficiency is reported in the paper.

^d)^ An indicator coded 1 if the paper’s publication outlet is a specialty journal of alternative medicine classified by MEDLINE.

^e)^ An indicator coded 1 if the paper is based on randomized controlled trials (RCT).

^f)^ A set of dummy variables indicating one of the NCCAM-generated five major modalities of alternative medicine [57] to which the specific treatment that the paper is testing belongs. Five modalities are “alternative whole medical system” involving acupuncture, Ayurveda, chelation therapy, traditional healers, alternative medical belief system, naturopathy, and homeopathy; “biologically based modalities” involving herbs, dietary supplements, vitamins/minerals, and special diets; “manipulation-based modalities” involving chiropractic, osteopathy, massage, anma, shiatsu, and movement therapies; “mind-body modalities” involving biofeedback, energy healing, hypnosis, yoga/taichi/qigong, relaxation, and psychotherapy; “other” involving spiritual/religious healing, self-care, and others.

Papers reporting practices in Japan are 2.07 times more likely than those in the U.S. to report the effectiveness of alternative medicine (*e*^0.731^ = 2.07 in Model 1). As hypothesized, a substantial part of this association is then explained by the deficiency of plurality coordination (Model 2). As soon as the U.S.–Japan difference in plurality coordination is taken into account, the relatively higher odds of Japanese studies finding alternative medicine to be effective reduce to 1.76 (= *e*^0.567^). This result remains unchanged even when Model 3 additionally controls for publication outlet, study design, and the modality of alternative medicine (see the Note in Table 3 for details of the variables). On the other hand, plurality coordination has a significant effect on treatment outcomes persistently across Models 2 and 3. When an alternative medicine treatment is found to be uncoordinated with biomedicine, it is less likely to produce effective treatment outcomes (odds ratio 0.12 = *e*^-2.117^).

Content analysis further provides qualitative evidence underlying this multivariate pattern. Many papers in the sample explicitly attribute ineffective and adverse treatment outcomes to coordination deficiency. First, unregulated and defective products of alternative medicine are held accountable. One article reports two patient cases in the U.S. that developed liver injuries by unwittingly taking bacteria-contaminated herbal supplements [98]. Practitioner commentaries argue that herbal medicine presents challenges to the medical community because the processes of cultivation, harvest, and manufacturing are not properly regulated for quality control [99, 100]. The 1994 U.S. Dietary Supplement and Health Education Act (DSHEA), which categorizes herbs and other botanicals as dietary supplements (and not drugs), was mostly criticized since it placed herbs outside of the regulation of the Food and Drug Acts [98, 101–104]. In the Japanese sample, early whirlpool bath-tubs are reported to have caused a significant number of people to drown while bathing, due to problems with the safety devices [105].

Second, the reports hold insufficient practitioner knowledge of alternative medicine responsible. A systematic review of 124 patients suffering adverse events from acupuncture in 89 medical reports finds that 85% of these adverse events resulted from the negligence, ignorance, or malpractice of acupuncturists who lacked proper education and training [106]. In the U.S., MDs and DOs practice acupuncture only with 220 hours of training and become a full member of the American Academy of Medical Acupuncture (AAMA) without board examinations [107]. In addition, clinical practice guidelines (CPGs) have mostly been found inadequate to guide physicians on how to cope with the use of alternative medicine in clinical settings [108].

A case report of a Cambodian immigrant woman in the U.S. shows that physicians’ unfamiliarity with alternative folk medicine that is often used among immigrant communities can result in an adverse treatment outcome [109]. The 73-year old Cambodian immigrant took a common blood thinner Warfarin after cardiac surgery for artificial heart valves. Later, she unexpectedly developed excessive bleedings recurrently for several years. Her doctor assumed that she did not comply with her medication or did not follow instructions to avoid a list of vitamin K foods that reportedly produce adverse interactions with Warfarin. As it turned out, however, the doctor was wrong. When the doctor had to visit the patient’s home because the patient declined to come to the hospital, he unexpectedly found that the patient was taking bitter melon which is high in vitamin K and was not on the doctor’s list of vitamin K foods. The patient was taking it from her own vegetable garden which is a common practice in her culture, concurrently with her prescribed medication. As soon as she stopped taking bitter melon, the bleeding problem was solved. The case report concludes with a note about the importance of plurality coordination through professional practitioners:

“Our clinical dietary advice had been based on our own expectations of food availability and consumption. What is natural to others was clearly not readily apparent to us. *Probing* for cultural or dietary practices rather than simply *prescribing* may have uncovered the secret of the bitter melon much earlier” (highlights added).

Third, the personal, informal, and commercial circulation of treatment information produces consequences as well. One article finds that, although the U.S. Dietary Supplement and Health Education Act regulated claims of “disease prevention, treatment, or cure” as unlawful, such claims were prevalent on commercial Internet sites of herbal supplements for cancers [110]. In this environment, consumers experienced difficulties in judging the quality of dietary products and services to treat obesity [111]. Another article stresses the importance of trained homeopathic practitioners, rather than misinformed self-care, in applying homeopathic drugs effectively [112]. Another article also finds that most of the Echinacea consumed in the U.S. is misused, based on its public misrepresentation as an effective cold and flu remedy, against its proven efficacy for treating infections [113].

At the same time, however, papers do report desirable synergic outcomes from the utilization of alternative medicine when it is coordinated with biomedical treatments. Treatment synergies have emerged from the text largely in three types. First, alternative medicine takes the place of conventional biomedical interventions that are less optimal, like acupuncture that was used for surgical analgesia that replaced drug analgesia producing perioperative side effects [114, 115]. Second, some modalities of alternative medicine complement biomedical interventions to the extent that their absence would make biomedical interventions impossible, such as herbs and acupuncture for bodily and sexual energy during chemotherapy and hormone therapies among cancer patients [37, 116] and religious/spiritual interventions to help patients to actively accept life even during extended biomedical treatments [41]. Third, alternative medicine is found to be simply additive to existing interventions, producing better outcomes. Several studies in Japan report the superior effectiveness of alternative medicine added to biomedical treatments, compared to the effectiveness of biomedical treatments alone, such as a Japanese herbal medicine (*Kampo*) added to Tamiflu for type A influenza [117] and acupuncture added to a conventional medication for chronic respiratory diseases (COPD) [36].

## Conclusions

Findings from the two complementary analyses have demonstrated that plural logics in medicine are consequential for health behavior and health care outcomes. When coordinated, the two different medical logics of biomedicine and alternative medicine have together generated resourcefulness for medical service users and practitioners, leading to desirable health care outcomes. When uncoordinated, on the contrary, the plural logics have generated strains for health behavior and led to undesirable outcomes. This result invites a reconsideration of existing models of plural action logics in medical sociology in particular, and those in organizational and institutional sociology in general.

The ethnographic notion about plural health behavior [52] that actors can somehow organize different elements of medical traditions within cultural orders should not overlook the fact that plural elements do not always co-exist easily. Even when these plural elements have come to an easy co-existence, this co-existence may most likely have come only after much difficulty and trouble, which is worth a close examination. This paper finds support for this argument from two recent efforts to re-conceptualize plural health behavior in terms of hybrid medical habitus, one among immigrant minorities in the U.S. [18] and the other among indigenous people in South Africa [19]. They both point to difficulty and trouble in plural health behavior, emphasizing the multiplicity and the incongruity of logics in plural medical systems.

While resonating with these two studies, this paper newly adds that there are significant variations in the behavioral consequences of multiple and incongruent plural logics, depending on how these uneasy and conflicting logics are managed. In this respect, this paper draws on a comparative cross-national insight that individuals subscribing to plural medical logics tend to have varying expectations of alternative medicine, depending on the institutional settings into which alternative medicine is practiced [81]. In this regard, this paper further demonstrates that it is not only the subjective user expectations but also the objective health care outcomes that are influenced by institutional settings.

Regarding the organizational and institutional sociology of plural action logics, this paper stresses that plural logics can have tensions with one another and actors are more or less skilled in dealing with these logics, depending on the social contexts in which plural logics and actors are situated. When it comes to the behavioral consequences of plural logics, therefore, this paper informs the simple resourcefulness account and the strains account of plural logics that it is problematic to monotonously assume either positive or negative consequences from plural logics. Instead, supra-individual social conditions need to be given analytical attention at multiple levels, such as government policies, insurance schemes, medical service organizations, and practitioners’ medical expertise and communication skills. Contingent upon these conditions, plural logics are open to generating both resourcefulness and strains for action.

To capture the significance of these conditions, this paper proposes plurality coordination as an important mechanism that generates the different behavioral consequences of plural logics. This proposition contributes to the existing literature in two ways. It provides a novel frame in which the divergent developments of plural logics between “settled” and “unsettled” times [13], between “senior” and “junior” scientists [11], between “resourceful” and “restrictive” museum environments [8], or between adolescents in “rich” and “poor” urban neighborhoods [12] that are discernible only to close readers in descriptive terms, can now be explicitly recognized and formally conceptualized. In addition, it also provides a meta-analytic perspective that can incorporate puzzling disagreements between empirical studies, for instance, why a similar set of plural logics are found to produce desirable outcomes in some study locales [38, 53] and not in others [19].

Lastly, this paper provides implications for the professional practice of medicine which in not a few countries has to engage with medical service users who rely on both biomedicine and alternative medicine at the same time. The findings suggest that the behavior of service users will be shaped by social contexts. Studies indeed agree by demonstrating how divergent the user behavior is in different institutional settings of healthcare system. The degree to which medical service users utilize both biomedicine and alternative medicine varies significantly between different healthcare systems [76]. The extent to which the users of alternative medicine adhere to its remedies also varies in different healthcare systems [75]. While awaiting future research that specifies how these behavioral variations lead to healthcare outcomes, practitioners of biomedicine as well as practitioners of alternative medicine need to be aware of the ways in which their healthcare systems affect the behavior of medical service users.

This paper has some limitations that future research needs to address. With regard to the cross-national panel data analysis, it uses proxy measures of medical resources for alternative medicine. This is inevitable given the scarcity of data in this field. Efforts need to be made in collecting more direct measures, such as the practitioner numbers of alternative medicine, equivalent to those of biomedicine. Regarding the content analysis of medical journal articles on alternative medicine, future research needs to expand the currently limited coverage of publication years (i.e. 1964 to 2009), in order to reflect developments in more recent medical trials and real-world practices. In a similar vein, efforts need to be made to collect live experiences of medical service users in plural medical systems that this paper is not able to apprehend in the cross-national panel or medical journal articles.

## Supporting information

S1 File Dataset1_panel analysis. (DTA)

S2 File Supporting text and tables. (DOCX)

S3 File Dataset2_content analysis. (DTA)
