# Supplementary material for: The coordination of plural logics of action and its consequences: Evidence from plural medical systems
Source: PLoS One. 2017 Dec 18;12(12):e0189841. doi: 10.1371/journal.pone.0189841 (PMC5734740; doi:10.1371/journal.pone.0189841)
Supplement: S2 File — (DOCX) [file pone.0189841.s002.docx]

**Supporting text and tables**

***For the coordination of plural logics of action and its consequences: evidence from plural medical systems***

**Text 1. Method note: medical resources in biomedicine and alternative medicine**

Direct measures of alternative medicine resources that are comparable to biomedical resources are not currently available. Some ideal measures would be the number of practitioners of diverse alternative medicine modalities, corresponding to the number of practicing medical doctors or nurses. The Yearbook serves as a source for alternative proxy measures. The international organizational memberships that a country has are found to be largely consistent with national organizational developments within the country (Paxton, 2002). The Yearbook has an established record of usage in the sociological literature on national and global organizational developments (Boli and Thomas, 1997; Beckfield, 2003). Some studies have also utilized the Yearbook’s existing 100 subject sections (with more sub-sections) to examine subject area-specific organizational developments in health/medicine (Inoue and Drori, 2006), economic exchange (Ingram et al., 2005), and uneven organizational developments in several social fields (Beckfield, 2010).

The medicine section lists 5,415 organizations in 2007, compared to 1,513 in 1985. To get a representative sample of these organizations, I have included all the organizations listed in one of the 25 sub-sections (i.e. “medicine”) for my analysis. This sub-section includes every medical organization whose title, aims, or activities explicitly mentions “medical.” This sample covers about 20% of all the medical organizations: 516 organizations in 1990 (24% of the total 2,131 medical organizations listed in the year); 536 organizations in 1995 (20% of the total 2,748); 603 organizations in 2000 (17% of the total 3,459). Out of these sample organizations, 228 organizations in 1990, 273 organizations in 1995, and 339 organizations in 2000 provide membership information (i.e. member countries). These organizations with membership information are finally used for computing the medical plurality index.

In distinguishing alternative medicine from biomedicine organizations, I have used 185 control vocabularies in the National Library of Medicine’s (NLM) Medical Subject Headings (MeSH) (Table A). I have compared the 185 terms with the titles, aims, activities, and regular publications of the organizations. Organizations that address at least one of the 185 MeSH terms are categorized into alternative medicine. Out of the organizations with membership information, 47 in 1990, 59 in 1995, and 68 in 2000 are alternative medicine organizations (its list available upon request from the author).

**Table A. 185 MeSH Terms for Alternative Medicine in the National Library of Medicine’s (NLM) Medical Subject Headings (MeSH)**

| Abreaction  Acoustic Stimulation  Acupressure  Acupuncture  Acupuncture Analgesia  Acupuncture Points  Acupuncture Therapy  Acupuncture, Ear  Ammotherapy  Animal Assisted Therapy  Anthroposophy  Apitherapy  Aromatherapy  Art Therapy  Association  Auriculotherapy  Autogenic Training  Autosuggestion  Aversive Therapy  Balneology  Baths  Behavior Therapy  Behavioral Medicine  Bibliotherapy  Biofeedback, Psychology  Breathing Exercises  Caloric Restriction  Catharsis  Chiropractic  Climatotherapy  Cognitive Therapy  Color Therapy  Complementary Therapies  Countertransference (Psychology)  Couples Therapy  Dance Therapy  Desensitization, Psychologic  Diabetic Diet  Diet Fads  Diet Therapy  Diet, Carbohydrate-Restricted  Diet, Fat-Restricted  Diet, Macrobiotic  Diet, Mediterranean  Diet, Protein-Restricted  Diet, Reducing  Diet, Sodium-Restricted  Diet, Vegetarian  Dietetics  Drainage, Sanitary  Eclecticism, Historical  Electroacupuncture  Environmental Health  Environmental Medicine  Equine-Assisted Therapy  Ethnopharmacology  Exercise Movement Techniques  Exercise Therapy  Eye Movement Desensitization Reprocessing  Faith Healing  Family Therapy | Feedback, Psychological  Feedback, Sensory  Free Association  Gestalt Therapy  Herbal Medicine  Holistic Health  Holistic Nursing  Home Health Aides  Homeopathy  Hydrotherapy  Hygiene  Hypnosis  Imagery (Psychotherapy)  Implosive Therapy  Integrative Medicine  Ketogenic Diet  Kinesiology, Applied  Laughter Therapy  Leeching  Magic  Magnetic Field Therapy  Manipulation, Chiropractic  Manipulation, Orthopedic  Manipulation, Osteopathic  Manipulation, Spinal  Marital Therapy  Massage  Medicine, African Traditional  Medicine, Arabic  Medicine, Ayurvedic  Medicine, Chinese Traditional  Medicine, East Asian Traditional  Medicine, Kampo  Medicine, Korean Traditional  Medicine, Mongolian Traditional  Medicine, Tibetan Traditional  Medicine, Traditional  Medicine, Unani  Meditation  Mental Healing  Meridians  Midwifery  Milieu Therapy  Mind-Body Relations (Metaphysics)  Mind-Body Therapies  Motion Therapy, Continuous Passive  Moxibustion  Mud Therapy  Musculoskeletal Manipulations  Music Therapy  Naturopathy  Nondirective Therapy  Nurse Midwives  Nutrition Therapy  Nutritional Support  Occultism  Organotherapy  Orthomolecular Therapy  Orthopedic Procedures  Osteopathic Medicine  Osteopathic Physicians  Palliative Care | Physical Medicine  Physical Therapy (Specialty)  Physical Therapy Modalities  Phytotherapy  Placebos  Play Therapy  Preventive Medicine  Psychoanalytic Therapy  Psychodrama  Psychological Techniques  Psychology, Medical  Psychophysiology  Psychosomatic Medicine  Psychotherapeutic Processes  Psychotherapy  Psychotherapy, Brief  Psychotherapy, Group  Psychotherapy, Multiple  Psychotherapy, Rational-Emotive  Radiesthesia  Reality Therapy  Reflexotherapy  Refuse Disposal  Rehabilitation  Rehabilitation Nursing  Rejuvenation  Relaxation Therapy  Residential Treatment  Resistance Training  Role Playing  Sanitary Engineering  Sanitation  Self Administration  Self Care  Self Medication  Sensitivity Training Groups  Sensory Art Therapies  Shamanism  Sleep Phase Chronotherapy  Social Medicine  Socioenvironmental Therapy  Sociology, Medical  Speleotherapy  Spiritual Therapies  Sports Medicine  Steam Bath  Suggestion  Superstitions  Tai Ji  Therapeutic Community  Therapeutic Touch  Tissue Therapy  Toilet Facilities  Transactional Analysis  Transference (Psychology)  Waste Disposal, Fluid  Waste Management  Water Purification  Water Supply  Witchcraft  Yoga |
| --- | --- | --- |

**References**

Beckfield J. (2003) Inequality in the World Polity: The Structure of International Organization. *American Sociological Review* 68: 401-424.

Beckfield J. (2010) The Social Structure of the World Polity. *American Journal of Sociology* 115: 1018-1068.

Boli J and Thomas GM. (1997) World Culture in the World Polity: A Century of International Non-Governmental Organization. *American Sociological Review* 62: 171-190.

Ingram P, Robinson J and Busch Marc L. (2005) The Intergovernmental Network of World Trade: IGO Connectedness, Governance, and Embeddedness. *American Journal of Sociology* 111: 824-858.

Inoue K and Drori GS. (2006) The Global Institutionalization of Health as a Social Concern. *International Sociology* 21: 199-219.

Paxton P. (2002) Social Capital and Democracy: An Interdependent Relationship. *American Sociological Review* 67: 254-277.

###### **Table B. Unstandardized Coefficients from the Random Effects Models of Life Expectancy Regressed on Medical Plurality Index and Control Variables**

|  | Model  1 | Model  2 | Model  3 | Model  4 | Model  5 | Model  6 | Model  7 | Model  8 |
| --- | --- | --- | --- | --- | --- | --- | --- | --- |
| Year Dummies (Reference = 1990) |  |  |  |  |  |  |  |  |
| 1995 | 1.11** | 0.91** | 0.87** | 0.90** | 0.44+ | 0.38 | 0.36 | 0.37 |
|  | (0.19) | (0.21) | (0.21) | (0.21) | (0.25) | (0.26) | (0.26) | (0.26) |
| 2000 | 2.28** | 1.87** | 1.84** | 1.85** | 0.94** | 0.83* | 0.80* | 0.82* |
|  | (0.22) | (0.27) | (0.27) | (0.28) | (0.34) | (0.36) | (0.36) | (0.36) |
| Medical Plurality Index (MPI) | -3.58+ | -2.65 | -4.50* | -2.89 | -2.88 | -2.53 | -3.40+ | -2.62 |
|  | (1.98) | (2.01) | (2.05) | (2.00) | (2.02) | (2.06) | (2.03) | (2.04) |
| Control Variables: Level of Organizational Memberships |  |  |  |  |  |  |  |  |
| Biomedicine Organizational Memberships |  | 0.15** |  |  |  | 0.05* |  |  |
| (per million people) |  | (0.03) |  |  |  | (0.02) |  |  |
| A.M. Organizational Memberships |  |  | 0.94** |  |  |  | 0.44** |  |
| (per million people) |  |  | (0.19) |  |  |  | (0.13) |  |
| Total Organizational Memberships |  |  |  | 0.14** |  |  |  | 0.05* |
| (per million people) |  |  |  | (0.02) |  |  |  | (0.02) |
| Control Variables: Socio-economic Conditions |  |  |  |  |  |  |  |  |
| GDP per capita (in hundreds) |  |  |  |  | 0.03** | 0.02** | 0.02** | 0.02** |
|  |  |  |  |  | (0.01) | (0.01) | (0.01) | (0.01) |
| Standardized Gini Coefficient of Income |  |  |  |  | -0.07 | -0.07 | -0.07 | -0.07 |
|  |  |  |  |  | (0.06) | (0.07) | (0.06) | (0.07) |
| Years of Education |  |  |  |  | 1.12** | 1.14** | 1.12** | 1.14** |
|  |  |  |  |  | (0.37) | (0.37) | (0.37) | (0.37) |
| Constant | 69.0** | 67.5** | 68.4** | 67.6** | 63.2** | 62.7** | 63.1** | 62.7** |
|  | (1.45) | (1.53) | (1.44) | (1.51) | (4.12) | (4.17) | (4.09) | (4.15) |
| Observations | 246 | 246 | 246 | 246 | 246 | 246 | 246 | 246 |
| Number of Countries | 97 | 97 | 97 | 97 | 97 | 97 | 97 | 97 |

*Note:* Robust standard errors in parentheses; + significant at 10%; * significant at 5%; ** significant at 1% (two-tailed tests).

**Table C. Unstandardized Coefficients from the Random Effects Models of Life Expectancy Regressed on Interaction Variables with Medical Plurality Index and Control Variables**

|  | Model 1 | Model 2 | Model 3 | Model 4 | Model 5^a)^ | Model 6^a)^ |
| --- | --- | --- | --- | --- | --- | --- |
| Year Dummies (Reference = 1990) |  |  |  |  |  |  |
| 1995 | 0.887** | 0.339 | 0.848** | 0.395 | 1.062** | 0.775** |
|  | (0.207) | (0.269) | (0.219) | (0.266) | (0.235) | (0.286) |
| 2000 | 1.891** | 0.830* | 1.773** | 0.853* | 2.117** | 1.505** |
|  | (0.273) | (0.382) | (0.295) | (0.372) | (0.288) | (0.370) |
| Medical Plurality Index (MPI) | -3.087 | -2.947 | -2.569 | -2.686 | -6.922* | -6.471* |
|  | (2.092) | (2.154) | (2.045) | (2.077) | (3.110) | (3.254) |
| Control Variables: Level of Organizational Memberships |  |  |  |  |  |  |
| Total Organizational Memberships | 0.123** | 0.057** | 0.145** | 0.046* | 0.105** | 0.051* |
| (per million people) | (0.027) | (0.021) | (0.030) | (0.023) | (0.031) | (0.021) |
| OECD18 Dummy | 8.948** | -3.171 |  |  |  |  |
| (1 for OECD18 countries; 0 for else) | (1.751) | (2.828) |  |  |  |  |
| MPI × OECD18 Dummy | 5.179* | 7.300* |  |  |  |  |
|  | (2.614) | (3.336) |  |  |  |  |
| Number of Medical Journal Papers (in thousands) |  |  | 0.126 | -0.057 |  |  |
|  |  |  | (0.123) | (0.069) |  |  |
| MPI × Number of Papers |  |  | -0.167 | 0.069 |  |  |
|  |  |  | (0.184) | (0.103) |  |  |
| Government Funding for A.M. |  |  |  |  | 5.084* | 0.714 |
| (1 for funding; 0 for else) |  |  |  |  | (2.490) | (2.675) |
| MPI × Funding for A.M. |  |  |  |  | 7.090+ | 7.690* |
|  |  |  |  |  | (3.814) | (3.848) |
| Control Variables: Socio-economic Conditions |  |  |  |  |  |  |
| GDP per capita (in hundreds) |  | 0.026** |  | 0.030** |  | 0.022** |
|  |  | (0.008) |  | (0.008) |  | (0.007) |
| Standardized Gini Coefficient of Income |  | -0.073 |  | -0.074 |  | -0.074 |
|  |  | (0.071) |  | (0.070) |  | (0.089) |
| Years of Education |  | 1.147** |  | 1.129** |  | 0.592 |
|  |  | (0.381) |  | (0.380) |  | (0.399) |
| Constant | 65.662** | 62.736** | 67.375** | 62.774** | 66.592** | 66.649** |
|  | (1.603) | (4.195) | (1.539) | (4.177) | (2.095) | (5.092) |
| Observations | 246 | 246 | 246 | 246 | 203 | 203 |
| Number of Countries | 97 | 97 | 97 | 97 | 76 | 76 |

*Note:* Robust standard errors in parentheses; + significant at 10%; * significant at 5%; ** significant at 1% (two-tailed tests); ^a)^ It is based on a subset of 203 observations with data on government funding for A.M.

**Table D. Unstandardized Coefficients from the Fixed Effects Models of Life Expectancy Regressed on Medical Plurality Index and Control Variables, among the Subset of 203 Observations**

|  | Model 1 | Model 2 | Model 3 | Model 4 |
| --- | --- | --- | --- | --- |
| Year Dummies (Reference = 1990) |  |  |  |  |
| 1995 | 1.423** | 1.373** | 1.600** | 1.625** |
|  | (0.183) | (0.203) | (0.305) | (0.320) |
| 2000 | 2.504** | 2.384** | 2.722** | 2.769** |
|  | (0.224) | (0.306) | (0.517) | (0.548) |
| Medical Plurality Index (MPI) | -7.106* | -7.009* | -5.944+ | -5.960+ |
|  | (3.000) | (2.990) | (3.483) | (3.494) |
| Control Variables: Level of Organizational Memberships |  |  |  |  |
| Total Organizational Memberships |  | 0.039 |  | -0.025 |
| (per million people) |  | (0.034) |  | (0.039) |
| Control Variables: Socio-economic Conditions |  |  |  |  |
| GDP per capita (in hundreds) |  |  | 0.013 | 0.016 |
|  |  |  | (0.008) | (0.010) |
| Standardized Gini Coefficient of Income |  |  | -0.023 | -0.024 |
|  |  |  | (0.087) | (0.088) |
| Years of Education |  |  | -0.626 | -0.645 |
|  |  |  | (0.863) | (0.876) |
| Constant | 73.937** | 73.613** | 77.069** | 77.239** |
|  | (1.670) | (1.662) | (6.350) | (6.452) |
| Observations | 203 | 203 | 203 | 203 |
| Number of Countries | 76 | 76 | 76 | 76 |
| R-squared | 0.49 | 0.49 | 0.51 | 0.51 |

*Note:* Robust standard errors in parentheses; + significant at 10%; * significant at 5%; ** significant at 1%.

**Table E. Unstandardized Coefficients from the Fixed Effects Models of Life Expectancy Regressed on Interaction Variables with Medical Plurality Index and Control Variables, among the Subset of 203 Observations**

|  | Model 1 | Model 2 | Model 3 | Model 4 | Model 5 | Model 6 |
| --- | --- | --- | --- | --- | --- | --- |
| Year Dummies (Reference = 1990) |  |  |  |  |  |  |
| 1995 | 1.347** | 1.563** | 1.389** | 1.673** | 1.254** | 1.427** |
|  | (0.205) | (0.330) | (0.212) | (0.328) | (0.213) | (0.352) |
| 2000 | 2.381** | 2.694** | 2.400** | 2.843** | 2.375** | 2.594** |
|  | (0.307) | (0.571) | (0.324) | (0.565) | (0.309) | (0.595) |
| Medical Plurality Index (MPI) | -7.755* | -6.752+ | -7.149* | -6.098+ | -9.903** | -8.862+ |
|  | (3.207) | (3.838) | (3.075) | (3.585) | (3.702) | (4.664) |
| Control Variables: Level of Organizational Memberships |  |  |  |  |  |  |
| Total Organizational Memberships | 0.038 | -0.027 | 0.039 | -0.045 | 0.040 | -0.016 |
| (per million people) | (0.033) | (0.037) | (0.036) | (0.046) | (0.033) | (0.034) |
| OECD18 Dummy^a)^ |  |  |  |  |  |  |
| (1 for OECD18 countries; 0 for else) |  |  |  |  |  |  |
| MPI × OECD18 Dummy | 7.268* | 6.470 |  |  |  |  |
|  | (3.564) | (4.112) |  |  |  |  |
| Number of Medical Journal Papers (in thousands) |  |  | -0.079 | -0.106 |  |  |
|  |  |  | (0.066) | (0.078) |  |  |
| MPI × Number of Papers |  |  | 0.120 | 0.132 |  |  |
|  |  |  | (0.103) | (0.122) |  |  |
| Government Funding for A.M.^a)^ |  |  |  |  |  |  |
| (1 for funding; 0 for else) |  |  |  |  |  |  |
| MPI × Funding for A.M. |  |  |  |  | 10.174* | 8.895+ |
|  |  |  |  |  | (4.243) | (5.033) |
| Control Variables: Socio-economic Conditions |  |  |  |  |  |  |
| GDP per capita (in hundreds) |  | 0.017+ |  | 0.020+ |  | 0.014 |
|  |  | (0.010) |  | (0.011) |  | (0.010) |
| Standardized Gini Coefficient of Income |  | -0.019 |  | -0.022 |  | -0.018 |
|  |  | (0.086) |  | (0.088) |  | (0.082) |
| Years of Education |  | -0.573 |  | -0.698 |  | -0.424 |
|  |  | (0.909) |  | (0.894) |  | (0.973) |
| Constant | 72.940** | 76.036** | 73.711** | 77.469** | 71.374** | 73.902** |
|  | (1.361) | (6.554) | (1.711) | (6.487) | (1.033) | (6.764) |
| Observations | 203 | 203 | 203 | 203 | 203 | 203 |
| Number of Countries | 76 | 76 | 76 | 76 | 76 | 76 |
| R-squared | 0.50 | 0.52 | 0.49 | 0.52 | 0.52 | 0.54 |

*Note:* Robust standard errors in parentheses; + significant at 10%; * significant at 5%; ** significant at 1%; ^a)^ Time-invariant indicator variables for countries are automatically dropped out of the FEM models.
